# Factors associated with food consumption score among pregnant women in Eastern Ethiopia: a community-based study

**DOI:** 10.1186/s41043-022-00286-x

**Published:** 2022-03-01

**Authors:** Meseret Belete Fite, Abera Kenay Tura, Tesfaye Assebe Yadeta, Lemessa Oljira, Kedir Teji Roba

**Affiliations:** 1 Department of Public Health, Institute of Health Sciences, Wollega University, Nekemte, Ethiopia; 2 School of Nursing and Midwifery, College of Health and Medical Sciences, Haramaya University, Harar, Ethiopia; 3 Department of Obstetrics and Gynaecology, University Medical Centre Groningen, University of Groningen, Groningen, The Netherlands; 4 School of Public Health, College of Health and Medical Sciences, Haramaya University, Harar, Ethiopia

**Keywords:** Prevalence, Food consumption score, Pregnant women, Ethiopia

## Abstract

**Introduction:**

Although assessing the nutritional status of pregnant women is becoming a common research agenda, evidence on food consumption scores, particularly among rural residents, is limited. This study aimed to assess the level of food consumption score and associated factors among pregnant women in Haramaya district, eastern Ethiopia, 2021.

**Methods:**

A community-based cross-sectional study was conducted among 448 pregnant women in Haramaya district, eastern Ethiopia. Data were collected through face-to-face interviews by trained research assistants, using a validated food frequency questionnaire. Food consumption score, a proxy measure for food security, was assessed through a seven-day dietary recall of consumption of food items. Each food item was given a score of 0–7 depending on the number of days it was consumed then grouped into food groups and summed up each food group. The pregnant women were labeled as "acceptable food consumption score" when they had a food composite score of > 42 during the reference period. Otherwise, they were defined as "unacceptable." A Poisson regression model with robust variance estimation was used to investigate the association of the independent variables with the food consumption score. An adjusted prevalence ratio with a 95% confidence interval was reported to show an association using a *p* value < 0.05.

**Results:**

The acceptable food consumption score among the study participants was 54.46% (95% CI 49–59%). The level of acceptable food consumption score was higher among respondents who were in the richest class (APR = 1.31; 95% CI 1.04–1.66), those who had ANC follow-up (APR = 1.78; 95% CI 1.40–2.27), those who had a favorable attitude toward dietary practice (APR = 1.30; 95% CI 1.12–1.52), and those who consumed high animal source foods (APR = 1.28; 95% CI 1.01–1.51). However, acceptable food consumption score was lower among women who were not owned agricultural land (APR = 0.84; 95% CI 0.72–0.99).

**Conclusion:**

We found a low acceptable food consumption score among pregnant women in this predominantly rural setting. Nutritional counseling in pregnancy should address the importance of food diversity and consumption of animal source foods to improve nutrition in pregnancy. Our findings highlight the imperative requirement for policies and programs to support farmers who had no farmland to improve their overall food security and maximize their food consumption score. Assessing perinatal outcomes associated with food consumption score is essential for unraveling the spectrum of nutrition in pregnancy and beyond.

## Introduction

Improved diet is believed to have a multiplier effect transversely to the Sustainable Development Goals (SDGs) [1]. The Food Consumption Score (FCS), a food frequency indicator developed by the World Food Programme (WFP) that intends to capture both diet quantity and quality of household food consumption [2]. Maternal food consumption during pregnancy is important since inadequate amounts of essential nutrients can adversely affect both mother and child. During pregnancy, the development of maternal tissues, fetal growth [3, 4], and breast milk production [5] increase nutritional requirements [6].

Ethiopia has endured fast urbanization with a swiftly advancing population [7]. It results that the food systems in rural areas may increase the apparent size and thus do the nutritional difficulty and the obtrusion required to break them. For instance, underprivileged rural communities are more reliant on food purchases and vulnerable to prices and other market shocks and hence to poor food consumption scores and under-nutrition [8]. Having sufficient food in terms of amount and quality is an essential constituent for a healthy and fertile existence [9, 10]. FCS is a composite score based on dietary diversity, food frequency, and relative nutritional importance of different food groups [11]. The available information confirming the relationship between food consumption and dietary practices arise from developed countries [12] founded on household measures of food consumption score as expressed by the head of the household. Although malnutrition decreased from 32.7 to 24.8% over the last two decades, sub-Saharan Africa has still the highest malnutrition [13, 14]. Ethiopia is also one of the countries where malnutrition is still extremely common [15].

In Ethiopia, various food security studies displayed that many households, particularly live in a predominantly rural setting, are food insecure. The question is not only just about food insufficiency but poor access to food [16, 17] and lacking in variety of dietary habits which is teff, wheat, corn, maize, and sorghum are the common staple food items in the study setting that are taken to poor food consumption [18]. Because of this poor food consumption both chronic and acute problems of food consumption are distributed and severe in both rural and urban settings of the country [7]. However, some study carried out on food insecurity which is a major indicator of food consumption score has stated more attention to the predominantly rural setting of the country do not establish context at the basic level. The prevalence of food consumption score during pregnancy was rarely documented in Ethiopia [19]. In this study, we assessed the level of food consumption score and associated factors among pregnant women.

## Methods

### Study settings

The study was embedded into the Haramaya Health Demographic Surveillance and Health Research Centre (HDS-HRC), which was established in 2018. The HDS-HRC located in Haramaya district. Haramaya District, located 500 km away from the capital city, Addis Ababa to the east. Haramaya district consists of 33 kebeles (the lowest administrative unit in Ethiopia). HDS-HRC covers 12 rural kebeles which is representative and randomly selected by considering geographic and environment issues. In HDS-HRC, 2306 pregnant women were followed. The district has mixed farming, with the major cash crop being khat (Catha edulis Forsk) [20]. The study was conducted from January 5 to February 12, 2021.

### Study design and population

A community based cross-sectional study was conducted. All pregnant women living in the district constituted the source population, whereas all pregnant women who lived in the selected kebeles for at least six months during the study period were the study population. Whereas those who consumed any food item which was not prepared at home and critically ill during data collection were excluded in this study. The sample size was determined using single and double population proportion formulas with their corresponding assumption, and the largest sample was considered. As such, the sample computed using single population proportion formula with the following assumptions gave the largest sample (*n* = 383): 95% confidence interval, level of acceptable food consumption score among pregnant women in Shegaw Motta (81.5%) [21], 5% marginal error and 10% non-response rate. However, this study is a part of the larger longitudinal study which obtained birth outcome information of pregnant women. Thus, the sample size used in this study was calculated from the larger study that included 475 pregnant women. After constructing the sampling frame from the HDS-HRC database, simple random sampling was applied to randomly select eight kebeles and then eligible women using a computer-generated lottery method.

### Data collection and measurement

Data were collected through interview administered questionnaires by trained research assistants. The questionnaire contained data on socio-economic, obstetric, maternal perception, food consumption, dietary knowledge, attitude, and practices of pregnant women. The questionnaire was initially prepared in the English language and was translated to the local language (Afan Oromo) by an individual with good command of both languages. It was also pre-tested on 10% of the sample in Kersa District before data collection. In addition, mid-upper arm circumference (MUAC) was measured to assess nutritional status.

Food consumption score is defined as the overall consumption of oil and sugars was frequent among all study participants, food consumption category of the women using the threshold; Poor food consumption score: 0 to 28, borderline food consumption score: 28.5–42 and Acceptable food consumption score: > 42 [22]. All consumption frequency of foods in the same group was summed and multiplied with value of each food group by its weight. To assess FCS, the participants were asked to recall the foods they consumed in the previous seven days before the survey. Each food item was given a score of 0 to 7 depending on the number of days it was consumed. Food items were grouped into food groups, and the frequencies of all the food items surveyed in each food group were summed. Any summed food group frequency value over 7 was recorded as 7. For each participant, the food consumption score was calculated by multiplying each food group frequency by each food group weight, then summed these scores into one composite score. Given the overall consumption of oil and sugars was frequent among all study participants, the food consumption category of the women using the threshold; poor food consumption score: 0 to 28, borderline food consumption score: 28.5–42, are categorized as unacceptable and Acceptable food consumption score: > 42.

The formerly validated food frequency questionnaire (FFQ) containing 27 of the most commonly lists of food items consumed by the district community was used to assess dietary diversity of the study participants [23–27]. Additionally, this validated FFQ was used to assess dietary diversity of the participants [28, 29]. Initially, the list of food items was established based on consultation of key informants living in the study area, who knew the culture, local language, and foods typically consumed. Then, the food frequency questionnaire was pretested on 10% of the sampled pregnant women in the district who were not included in the main study and necessary modifications were made based on the observations. In addition, pretested food frequency questionnaires were carried out on 10% of the sampled pregnant women of the district not included in the main study. Necessary modifications were made before actual implementation to generate data. Finally, to measure the consumption of each food per day, per week or per month for the FFQ in the past three months to consider the difference of dietary consumption within a day of a week to take the concept into account. However, we considered the greater difference of dietary practice in the local community over the day of the week, the intake of each food item per day [30, 31] was not taken as a cut-off point to label consumers. In doing so, pregnant women were defined as “consumer” of a food item if they had consumed those items at least once over a period of a week [28, 32].

The food items in the FFQ were grouped into ten food groups. These are: cereal, white roots and tubers, pulse and legumes, nuts and seeds, dark green leafy vegetables, other vitamin A-rich fruits and vegetables, meat, fish and poultry, dairy and dairy product, egg, other vegetables, and other fruits [33]. The sum of each food group that the pregnant women consumed over a period of one week were calculated to analysis the dietary diversity score (DDS). Furthermore, dietary diversity score was converted into tertiles, and the highest tertile used to label “high” dietary diversity score whereas both lower tertiles combined were defined as” low” dietary diversity score. Food variety score (FVS) is the frequency of individual food items consumed in the reference period of the study. Therefore, it was estimated by the intake of 27 food items by each individual over seven days [28], with maximum of FVS fourth. Finally, the mean FVS of pregnant women was calculated and those of them with FVS greater than the means were labeled as having “high” food variety score, whereas those with FVS lower than the means were defined as having “low” FVS. Furthermore, consumption of foods from animal source (ASF) was estimated by counting the frequency of each food from animal sources that pregnant women ate over a reference period. Animal source foods score was also converted into tertile and the highest tertile used to label as “highs, while the two lower tertile combined were defined as “low” ASF.

### Data quality assurance

Two days of rigorous and extensive training with the final version of the questionnaires was given to each data collector and supervisor prior to pre-test. Collected data were checked by supervisors before being sent to the data entrée on daily basis. We pre-tested the questionnaires on 10% of the sampled pregnant women of the Kersa district, that were not included in the main study, and modification was done based on the pre-test observations. The supervisors kept the alleyway of the field procedures and checked the completed questionnaires daily to approve the accuracy of data collected, and the research team managed the overall work of data collection.

### Data processing and analysis

Data were double entered using EPiData version 3.1 software. Data were cleaned, coded, and checked for missing and outliers, for further analysis exported to STATA version 14 (College Station, Texas 77845 USA) statistical software. The outcome variable was dichotomized as food consumption score = 1 (Acceptable) and food consumption score = 0 (Unacceptable). Thus, the Poisson regression analysis model with robust variance estimate was fitted to identify predictors of the food consumption score of women. For multivariable analyses, only variables that displayed a *p* < 0.25 in the bivariate analyses were entered in the adjusted model. The backward regression was fitted with selected socio-economic and fertility-related variables. The results are presented as adjusted prevalence ratios (APRs) with 95% CI. The statistical level of significance was set at alpha = 5%. Possible interactions between covariates were tested. Akaike's information criterion (AIC) and Bayesian information criterion (BIC) were used to test for model fitness. All variables with *p* < 0.25 in the bivariate analyses were included in the final model of multivariable analysis to control all possible confounders. Multicollinearity test was carried out to see the correlation between independent variables by using the standard error and collinearity statistics (variance inflation factors > 10 and standard error > 2 were considered as suggestive of the existence of multi co-linearity). Correlation between independent variables was checked using the Pearson Correlation Coefficient.

To estimate the economic level of the families, a wealth index was employed. The wealth dispersion was generated by applying principal component analysis. The index was calculated based on the ownership of latrine, selected household asset, quantity of livestock, and source of water used for drinking, that was to 41 household variables. Nutritional knowledge of the women was gauged through 16 nutritional knowledge questions on the feature of nutrition needed in their course of pregnancy. Lastly, the highest tertile was defined as having "Good" nutritional knowledge, and the two lower tertiles were labeled as "Poor" nutritional knowledge. The maternal attitude was evaluated with 12 Likert scale questions using PCA. The factor scores were totaled and classified into tertiles (three parts), and the highest tertile was defined as having a "Favorable" maternal attitude, and the two lower tertiles were characterized as "Unfavorable" maternal attitude. The maternal perceived vulnerability of malnutrition was evaluated with 10 Likert scale questions using PCA. The factor scores were totaled and classified into tertiles (three parts), and the highest tertile was defined as having a perceived vulnerability "Yes," and the two lower tertiles were characterized as "No" maternal perceived vulnerability. Similarly, perceived severity of malnutrition, perceived benefit to healthy nutrition perceived barrier to healthy nutrition, and perceived self-efficacy to control malnutrition during pregnancy were calculated by using their composite questions. Women's autonomy was evaluated by seven validated questions which were adopted from the Ethiopian demographic health survey [29]. For each response to a question, the response to each question was coded as "one" when the decision was made by the pregnant women alone or jointly with their husband, otherwise "zero."

### Ethical consideration

All methods of this study were carried out in accordance with the Declaration of Helsinki—Ethical principle for medical research involving human subjects. An ethical approval letter was obtained from Haramaya University Institutional Research Ethics and Review Committee (IRERC) with a reference number of (IHRERC/266/2020) before the commencement of data collection. Written informed consent to participate was obtained from participants and legally authorized representatives "of minors below 16 years of age and illiterates," and their privacy and confidentiality were maintained. All personal identifiers were excluded, and data were kept confidential and used for the proposed study only.

## Results

### Socio-demographic characteristics

A total of 475 pregnant women were eligible, 448 consented, making a response rate of 94.3%. The mean age of the women was 25.68 (± 5.1), ranging from 16 to 36. The majority of the respondents could not read or write (73.88%), were housewives (96.1%), farmers (93%), and had a family size of 1–5 (76.56%). Only 20.09% of the respondents were in the richest quintiles, Table 1.

**Table 1:** Socio-demographic of pregnant women in Haramaya District, eastern Ethiopia, 2021 (*n* = 448)

Variables	Frequency (*n*)	Percentage (%)
Age (years)		
< 18	25	5.58
18–35	400	89.29
> 35	23	5.13
Mean (± SD)	25.68 (± 5.16)	
Educational level of the woman		
Can’t read or write	331	73.88
Read or write	26	5.81
Formal education	91	20.31
Educational level of husband		49 (23.33)
Can’t read or write	259	57.81
Read or write	61	13.62
Grade 1–8	102	22.77
Grade 9 and above	26	5.8
Occupation of the woman		
Housewives	433	96.65
Merchants	15	3.65
Occupation of husband		
Farmers	420	93.75
Daily labors	28	6.25
Family size		
1–5	343	76.56
≥ 5	105	23.44
Agricultural land possession		
No	271	60.49
Yes	177	39.51
Livestock possession		
Yes	299	66.74
No	149	33.26
Wealth index (quintile)		
Poorest	90	20.09
Poor	90	20.09
Middle	89	19.87
Rich	90	20.09
Richest	89	19.87
Parity		
0	103	22.99
1–4	294	s65.63
≥ 5	51	11.38

### Food consumption scores

**Fig. 1:**
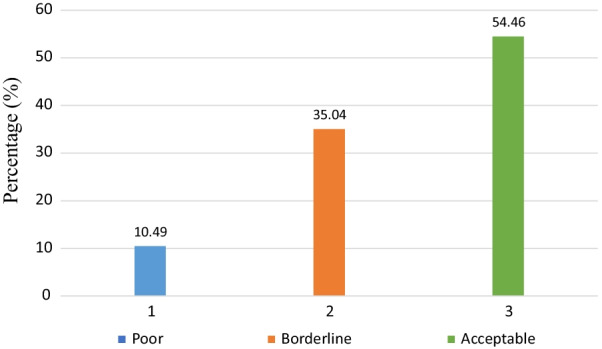
Food consumption score of pregnant women in Haramaya District, eastern Ethiopia, 2021

The proportion of pregnant women's food consumption scores before the study period is presented in Fig. 1. The acceptable food consumption score among the study participants was 54.46% (95% CI 49–59%). Of the respondents, 10.49% and 35.04% had a poor and borderline food consumption scores, respectively. From the total respondents, 29.46% (25–34%), 37.5% mean (± SD) (9.03 ± 2.79) and 24.78% (2–29%) of them had high dietary diversity, high food variety score and high consumption of animal source food, respectively, Table 2.

**Table 2:** Dietary practices of pregnant women in Haramaya district, eastern Ethiopia, 2021

Variables	Frequency (*n*)	Percentage (%)
Dietary diversity score (DDS)		
Low	316	70.54
High	132	29.46
Mean (± SD)	3.73 (± 1.33)	
Food variety sore (FVS)		
Low	280	62.50
High	168	37.50
Mean (± SD)	9.03 (± 2.80)	
Animal source foods (ASFs)		
Low	337	75.22
High	111	24.78
Mean (± SD)	25.68 (± 5.16)	
Meal frequency		
< 4	331	73.88
≥ 4	117	26.12
Mean (± SD)	2.98 (± 0.84)	

### Factors associated with food consumption score

In the crude analysis wealth, agricultural land possession, ANC visit, attitude, meal frequency, Consumption of ASFs, Craving (strongly liked for food), educational level of women, educational level of husband, occupational status of husband and women decision making were found to be a candidate for multivariable analysis at *p* < 0.025. In the adjusted analysis, only wealth, ANC follow-up, consumption of animal source foods, agricultural land possession, and attitude remained statistically significant. The level of FCS was higher among respondents who were in the richest class (APR = 1.31; 95% CI 1.04–1.66), those who had ANC follow-up (APR = 1.78; 95% CI 1.40–2.27), those who had a favorable attitude toward dietary practice (APR = 1.30; 95% CI 1.12–1.52), and those who consumed high animal source foods (APR = 1.28; 95% CI 1.01–1.51). However, acceptable food consumption score was lower among women who were not owned agricultural land (APR = 0.84; 95% CI 0.72–0.99) (Table 3).

**Table 3:** Factors associated with food consumption score among pregnant women in Haramaya District, eastern Ethiopia, 2021 (*n* = 448)

Variables	Food consumption score		CPR (95%CI)	APR (95%CI)	*p* value
	Acceptable (*n* = 244)	Unacceptable (*n* = 204)			
Wealth index (quantile)					
Poorest	48 (19.67)	42 (20.59)	1	1	
Poor	*50 (20.49)*	40 (19.61)	10.4 (0.79, 1.36)	1.78 (.91, 1.52)	0.021 *
Middle	42 (17.21)	47 (23.04)	0.88 (0.66, 1.12)	1.05 (0.77, 1.42)	
Rich	40 (16.39)	50 (24.51)	0.83 (0.62, 1.13)	1.01 (0.77, 1.35)	
Richest	64 (26.23)	25 (12.25)	1.35 (1.07, 1.70)	1.31 (1.04, 1.66)	
Agricultural land possession					
Yes	107 (43.85)	70 (34.31)	1	1	
No	137 (56.15)	134 (65.69)	0.83 (0.70, 098)	0.84 (0.72, 0.99)	0.046*
ANC visit					
No	53 (21.72)	111 (54.41)	1	1	
Yes	191 (78.28)	93 (45.59)	2.08 (1.64, 2.64)	1.78 (1.40, 2.27)	< 0.001**
Attitude					
Unfavorable	135 (55.33)	167 (81.86)	1	1	
Favorable	109 (44.67)	37 (18.14)	1.67 (1.43, 1.95)	1.30 (1.12, 1.52)	< 0.001**
Meal frequency					
< 4	156 (63.93)	175 (85.78)	1	1	
≥ 4	88 (36.07)	29 (14.22)	1.60 (1.36, 1.86)	1.16 (0.94, 1.43)	0.158
Consumption of ASFs					
Low	162 (66.39)	175 (85.78)	1	1	
High	82 (33.61)	29 (14.22)	1.54 (1.31, 1.79)	1.22 (1.01 1.51)	0.049*
Craving (strongly liked for food)					
No	114 (54.55)	126 (72.41)	1	1	
Yes	95 (45.45)	48 (27.59)	0.72 (0.60, 0 .85)	0.82 (0.67, 0.95)	0.231

*CPR* crude prevalence ration, *APR* adjusted prevalence ratio

**Statistically significant at *p* value < 0.001; *Statistically significant at *p* value < 0.05

## Discussion

The purpose of this study was to assess the level of food consumption score and its determinants among pregnant women in Haramaya District, eastern Ethiopia. We found that only slightly more than half (54.7%) pregnant women had acceptable FCS. Factors include Wealth, ANC follow-up, consumption of animal source foods, agricultural land possession, and attitude were positively associated with acceptable food consumption score.

Our finding is lower than studies conducted in different parts of Ethiopia [21, 32], Mali [33] and study of World Food Program [22]. Our finding is, however, higher than findings from studies conducted in Niger, Somalia, Burundi, Uganda and Sudan [34–37]. This might be due to poor food availability, utilization of diet, inappropriate dietary practice and less common intake of animal source foods in our study area. Additionally, geographical variation and methodological difference, socio-economic, cultural settings and seasonal differences might contribute to this difference. We found that antenatal care, attitude toward dietary practice, consumption of animal source foods, wealth status, and restriction of intake of some foods were factors associated with acceptable FCS. In agreement to our result, different epidemiological studies reported that attitude is the significant factor associated with level of FCS in pregnancy [38–40].

In the current study, food consumption score was observed greater among pregnant women who consumed ASFs. In Ethiopia, the government is developing an accelerated stunting reduction strategy which is principally centering on food-based approaches. Information displays that animal ownership has possibility for economic benefits and share to ASF intake in poor communities [28]. There is a demand for innovative ways of improve both local manufacture and enhanced intake of ASF by pregnant women at the household level. A finding from the highlands of the country displayed that collaborated approach for the scale up of local dairy goats and cows managed by women with an aggregation of improved administration method, genetic improvements' guide to good access of households to ASFs [41].

Finding ways to better food security of small farmer holders and no farm land is critical development goals in numerous developing countries [42], as they are responsible for much of farm production and are also the most food insecure and poorest population and hence targets of endeavor to last hunger and relieve poverty. In the present study, food consumption score was observed greater among pregnant women who had land farming. In present study, who owned agricultural land in line with study conducted in Nigeria [43], Malawi [4] and Central America [45]. Our findings highlight the imperative requirement for policies and programs to support farmers who had no farm land to improve their overall food security and maximize shier food consumption score. Such policies must focus on intensifying household education levels, securing land tenure, encourage women, promoting generational knowledge conversation, and supplying emergency food assistance. Not unexpectedly, household economic status was positively significantly associated with food consumption score of pregnant women. A similar association was previously reported in Illu Aba Bor Zone, Southwest Ethiopia [46].

Antenatal care service gives an essential chance for discourse between a pregnant women and a health care workers about health behavior during pregnancy and about realizing complications that may come up during pregnancy [47]. Disregard important improvements in the coverage of ANC over the last two decades, about 40 of women worldwide still do not get the suggested four regular ANC visits [48], which could be due to interaction between possible factors such as rural residence low maternal education, and poverty across the region of sub-Saharan Africa [49]. Numerous women do not begin ANC early enough to exploit all chance for ANC, perhaps due to lack of knowing or content or misconceptions of the intention and worth of ANC, the proper period of time to get ANC, expedited approval of pregnancy, and/or absence of assistance from partner [50].

The strength of our study was the use validated food frequency questionnaire for assessing dietary practice. Food items were established based on wide-ranging consultation with key informants who are from the study area and who knew the culture and local language on the types and food groups usually consumed. Our study also has some limitations that should be taken into consideration. First, the cross-sectional nature of the data limits causal inference between FCS and their correspondences, and due to sample collection being from a single season, this limits the generalizability of the results to other reasons. Second, since there is individual difference of dietary consumption over the period of seven days. Third, women who reported consumption of more than one food items in the seven days were also tagged with those who consumed only once. Finally, FCS is a good indicator of a household's food security; however, it does not help with understanding the quality of diets consumed by a specific group of household members, such as children 6–59 months of age.

## Conclusion

The study showed that the level of acceptable FCS of pregnant women in the Haramaya district is sub-optimal. ANC follow-up, attitude toward dietary practice, wealth, and consumption of animal source foods were factors independently associated with having acceptable FCS. Nutritional counseling in pregnancy should address the importance of food diversity and consumption of animal source foods to improve nutrition in pregnancy. Production and increased intake of animal source foods, like the egg, by pregnant women at the household level are essential to achieve acceptable FCS. Our findings highlight the imperative requirement for policies and programs to support farmers who had no farmland to improve their overall food security and maximize their food consumption score. Assessing perinatal outcomes associated with food consumption score is essential for unraveling the spectrum of nutrition in pregnancy and beyond.

There is a need to examine effect of FCS improving perinatal outcomes.

## Data Availability

All data are available within the manuscript. Additional data can be obtained from the corresponding author on a reasonable request.

## Abbreviations

ANC: Antenatal care
ASF: Animal source food
DDS: Dietary diversity score
EDHS: Ethiopian Demographic and Health Survey
FCS: Food consumption score
FVS: Food variety score
HDS-HRC: Health Demographic Surveillance and Health Research Center
PCA: Principal component analysis
WHO: World Health Organization
